# Metabolite-based mutualism enhances hydrogen production in a two-species microbial consortium

**DOI:** 10.1038/s42003-019-0331-8

**Published:** 2019-02-28

**Authors:** Shaojie Wang, Hongzhi Tang, Fei Peng, Xijia Yu, Haijia Su, Ping Xu, Tianwei Tan

**Affiliations:** 1 Beijing Advanced Innovation Center for Soft Matter Science and Engineering, and Beijing Key Laboratory of Bioprocess, Beijing University of Chemical Technology, 100029 Beijing, P. R. China; 2 State Key Laboratory of Microbial Metabolism, and School of Life Sciences & Biotechnology, Shanghai Jiao Tong University, 200240 Shanghai, P. R. China

## Abstract

Sustainable hydrogen production from renewable and low-cost substrates is very important to mitigate environmental and energy-related issues. Microbial consortia are promising for diverse bioenergy and environmental applications, yet microbial interactions are not fully understood. Here, we present comprehensive investigation on how two species in an artificial microbial consortium, consisting of *Bacillus cereus* A1 and *Brevundimonas naejangsanensis* B1, mutually cooperate to achieve an overall enhancement in hydrogen production and starch utilization. In this consortium, strains A1 and B1 secrete α-amylase and glucoamylase that are functionally complementary in starch hydrolysis. Moreover, strain A1 converts starch into lactate as a carbon source and electron donor, supporting the cell growth and hydrogen generation of strain B1. In return, strain B1 produces formate as an electron shuttle to strain A1 to enhance hydrogen production. The co-culture re-directs the overall metabolic flux, facilitates the cell growth, and up-regulates the key genes of hydrogen production and starch hydrolysis.

## Abstract (web summary)

Wang and Tang et al. show that two-species microbial consortium is more efficient in hydrogen production and starch utilization, compared to pure cultures. This work underscores a utility of synthetic microbial consortia, which can be optimized for increasing production of a certain metabolite.

## Introduction

Mounting concerns about traditional sources and environmental pollution has promoted the extensive researches on clean and renewable energy^1,2^. Hydrogen is considered as the most promising energy source, with the highest energy content and no CO_2_ emission^3^. Currently, hydrogen is mainly produced by chemical methods such as steam reforming of hydrocarbons and gasification of biomass^4,5^. Biological hydrogen production is an attractive approach because it can use a wide variety of low-cost renewable materials^6,7^. Starch is considered as a cost-effective substrate for biohydrogen production^8,9^. Amylose and amylopectin are two major components of starch, which represent almost 98–99% of starch dry weight^10^. Amylose is a linear polymer and amylopectin is highly branched^11^. These polymers naturally occur as condensed and insoluble granules with semicrystalline regions, hampering starch hydrolysis by a pure bacterial culture^10,12^. Several attempts have been made to increase hydrogen production by enhancing the process of starch hydrolysis such as starch pretreatment by heat^13^ or enzyme-based digestion^14^ and separating the steps of starch hydrolysis and hydrogen production^15^. Nevertheless, pretreatment as well as the subsequent steps for separation requires additional processing, thereby increasing costs.

Microbial consortia are ubiquitous in nature and are widely used for a variety of important processes^16–18^, due to their high adaptability, broad substrate spectra, and the possibility of continuous processes^17^. Compared to pure cultures, microbial consortia usually possess a larger pool of genes and more diverse metabolic pathways and use less refined substrates (such as molasses, raw starch, etc.)^19,20^. Furthermore, microbes in a consortium can coordinate their specific activities by trading metabolites or exchanging signals^19^. However, because of the complex microbial composition of a natural consortium, it is less stable, thus making it difficult to scale up the processes, greatly restricting their practical applications because of unknown genetic backgrounds of many wild-type species^21,22^.

Synthetic or artificial microbial consortia, with a defined composition and controllable functions, offer a promising approach to promote operational stability, substrate utilization, and production yields^19,20^. Because microbes in artificial consortia have been selected to perform particular tasks, the applications are more specific with high efficiencies than nature consortia. However, construction of artificial microbial consortia requires a detailed and comprehensive understanding of molecular mechanisms underlying cell–cell interactions^19^. Although a number of studies have been conducted to characterize the specie diversity in different natural consortia from the oceans to the human gut, we still lack of a clear understanding of fundamental molecular and ecological bases of community-level functions and the potential cell–cell interactions^22,23^.

The interactive mechanisms in microbes can be divided into two types, i.e., contact-independent and contact-dependent interactions. In the contact-independent interactions, microorganisms interact with each other by exchanging different metabolites and information signals. Marine bacterium *Vibrio fischeri* regulates its bioluminescence through a quorum sensing mechanism by accumulating autoinducer in the environment as the population density increases^24–27^. Microbes can also establish cell–cell interactions by different metabolites, including small molecules and large molecules^28,29^. For example, in a synthetic three-species microbial consortium for bioelectricity generation, *Escherichia coli* and *Bacillus subtilis* could convert glucose into lactate and riboflavin for *Shewanella oneidensis* to generate electricity, while *S. oneidensis* could produce acetate as the carbon source for *E. coli* and *B. subtilis*^21^. In the direct contact-dependent interactions, microorganisms exchange electrons by direct interspecies electron transfer^30^ or deliver macromolecules (such as DNA and proteins) through conjugation^31,32^. Rotaru et al.^30^ found that *Geobacter metallireducens* could convert ethanol to methane and directly transfer electrons to *Methanosaeta harundinacea* via its conductive pili, and *M. harundinacea* could accept electrons for the reduction of carbon dioxide to methane.

Previously, we isolated hydrogen-producing bacteria from anaerobic activated sludge, i.e., *Bacillus cereus* A1^33^ and *Brevundimonas naejangsanensis* B1^34^. We used these two strains to form an artificial microbial consortium for hydrogen production and found that the consortium enhanced hydrogen production as well as starch utilization compared to that using pure culture. Hereby we comprehensively investigate bacterial interactions and illuminate how the two species work mutually to achieve an overall enhancement in hydrogen production and starch utilization via multi-omics method. This study may provide insights for designing more complex synthetic microbial consortia for their applications.

## Results

### Enhanced hydrogen production by the microbial consortium

**Fig. 1:**
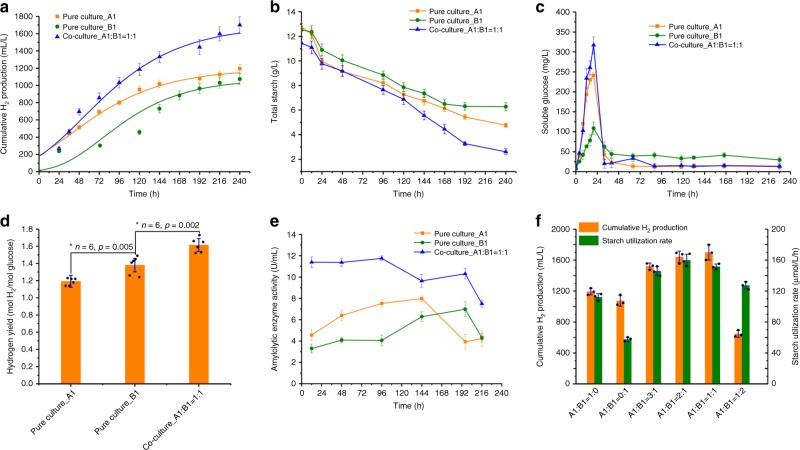
The performance of hydrogen production from starch between pure cultures and co-culture at a mixed ratio of A1:B1 = 1:1. **a** Cumulative hydrogen production, **b** total starch consumption, **c** reducing glucose production, **d** hydrogen yield, and **e** extracellular amylolytic enzyme activities. Two bacteria, *Bacillus cereus* A1 and *Brevundimonas naejangsanensis* B1, were used in this study. Cumulative hydrogen production was fitted by a modified Gompertz model. Error bars indicate standard deviations from at least three repeat experiments. Significant difference between different groups represent *p < 0.05. **f** Effects of different mixed ratios on cumulative hydrogen production and starch utilization rate

In this study, we constructed an artificial two-species microbial consortium that was highly efficient for hydrogen production from corn starch. As shown in Fig. 1a, this two-species microbial consortium could produce 1698.5 ± 97.1 mL L^−1^ hydrogen, which was 42.3% and 58.2% higher than that of the pure cultures of strain A1 and strain B1, respectively. Pure culture of strain A1 or strain B1 could hydrolyze 62.7% and 49.9% of total starch, respectively, while co-culture consortium could improve the hydrolysis efficiency up to 77.4% (Fig. 1b). Further analysis showed that starch was hydrolyzed into glucose within 20 h but was quickly utilized afterwards (Fig. 1c). Pure culture of strain B1 showed a poor starch-hydrolyzing ability, with a maximum glucose production of 108.8 ± 15.3 mg L^−1^, while pure culture of strain A1 could produce 240.9 ± 7.9 mg L^−1^ glucose within the same time. In the co-culture, we found that glucose increased to 317.1 ± 20.6 mg L^−1^, an almost three-fold increase compared with the pure culture of strain B1. Although strain A1 exhibited a better starch-hydrolyzing ability than strain B1, the final hydrogen yield of pure strain A1 culture was much lower than that of pure strain B1 culture (1.19 vs. 1.38 mol H_2_ per mol glucose), indicating that strain B1 is more efficient for hydrogen production than strain A1. Notably, the co-culture could increase the hydrogen yield to 1.61 mol H_2_ per mol glucose (Fig. 1d), which may benefit from the mutual interactions of these two-species microbial consortium.

We then determined the crude amylolytic enzyme activity in pure cultures and co-culture in vitro (Fig. 1e). Before 96 h, the amylolytic activity of pure culture of strain B1 remained at a low level of 4.1 ± 0.5 U mL^−1^, while pure culture of strain A1 showed an increasing amylolytic activity with the maximum enzyme activity of 7.5 ± 0.4 U mL^−1^, more than two-fold higher than the pure culture of strain B1. However, the enzyme activity of strain B1 continuously increased to 7.0 ± 0.7 U mL^−1^ after 144 h, while that of strain A1 decreased to 3.9 ± 0.7 U mL^−1^. These results suggest that strain A1 might play an important role in starch hydrolysis at early stage while strain B1 was more important at later stage. However, during the entire fermentation process, the co-culture exhibited much higher amylolytic activity (9.6–11.6 U mL^−1^) compared to the pure cultures, further indicating that the co-culture of these two strains can enhance the starch hydrolysis process.

Further, we tested the effects of different mixed ratios on hydrogen production and starch utilization (Fig. 1f). Strain B1 showed a lower starch hydrolysis ability than strain A1, but the co-culture, at a mixture ratio of 1:1, increased the starch utilization rate by 34.9% and 163.6%, respectively, compared to the pure culture of strain A1 and strain B1. Co-culture at different ratios showed a higher starch utilization rate than the pure cultures, and the highest starch utilization rate was obtained at an A1:B1 ratio of 1:1 and 2:1 (v/v), with the highest hydrogen production of 1698.5 ± 97.1 mL and 1640.8 ± 159.6 mL, respectively. However, a lower fraction of strain A1 (A1:B1 = 1:2) decreased the starch utilization rate by 20.3% and only generated 644.3 ± 127.2 mL L^−1^ hydrogen, a 59.0% reduction compared with A1:B1 = 1:1 or 2:1. Nevertheless, a higher fraction of strain A1 (A1:B1 = 3:1) did not enhance the extent of starch utilization but slightly decreased hydrogen production by 11.7% (Fig. 1f).

### Genomic analysis and metabolic identification of the microbial consortium

**Fig. 2:**
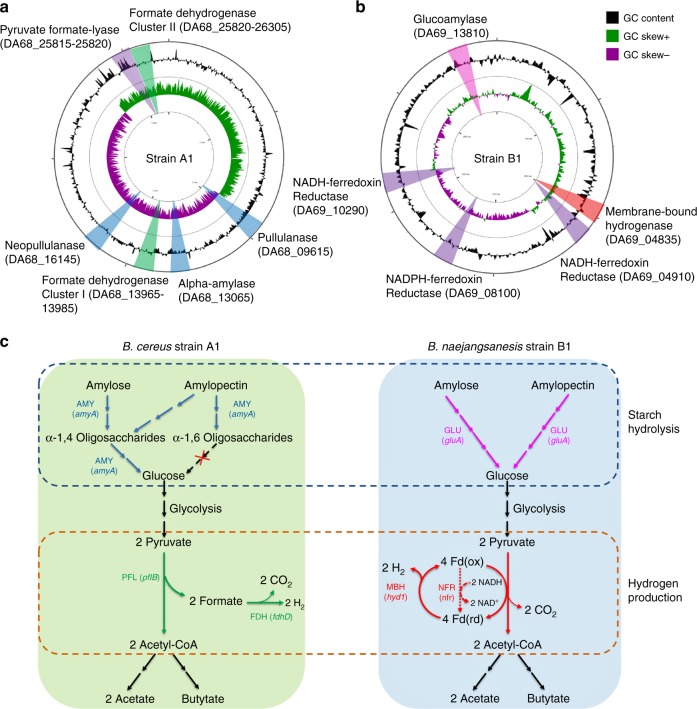
Genome maps of **a** *Bacillus cereus* A1 and **b** *Brevundimonas naejangsanensis* B1. Circle 1 indicates the value of GC skew (G−C/G+C). Circle 2 indicates the percentage of GC content. Hydrogen production and starch hydrolysis-related genes are marked on the maps. Strain A1 possesses three starch hydrolysis-related genes (marked in blue sector), encoding pullulanase, neopullulanase, and α-amylase. Strain A1 also harbors two formate dehydrogenase clusters (marked in green sector), and a pyruvate formate-lyase encoding gene (marked in purple sector), indicating hydrogen is produced by formate cleavage. Strain B1 has one starch hydrolysis-related gene (marked in pink sector), encoding glucoamylase. In addition, strain B1 has a membrane-bond hydrogenase (marked in red sector) and three NAD(P)H-ferredoxin reductases (marked in purple sector), suggesting hydrogen is produced from reduced ferredoxin. **c** Simplified metabolism pathway for hydrogen production and starch hydrolysis in strains A1 and B1. AMY α-amylase, GLU glucoamylase, FDH formate dehydrogenase, NFR NAD(P)H-Ferredoxin reductase, MBH membrane-bound hydrogenase. The corn starch in the present study consists of approximately 27% amylose and 63% amylopectin

To explore the synergistic interactions between strains A1 and B1 in the co-culture, the two strains were subjected to whole-genome sequencing. The genome maps of strains A1 and B1 are presented in Fig. 2a, b. The genome sequences of strains A1 and B1 were further subjected to automated analysis by Rapid Annotation using Subsystem Technology^35^. The key genes involved in starch hydrolysis and hydrogen production are indicated on the genome maps (Supplementary Table 1). The metabolic pathways of strains A1 and B1 were further analyzed using the Kyoto Encyclopedia of Genes and Genomes database^36^. Based on the above information, simplified metabolic pathways for hydrogen production from starch by strains A1 and B1 were constructed (Fig. 2c).

Strain A1 genome possesses two genes encoding pullulanase (DA68_09615) and neopullulanase (DA68_16145) for starch hydrolysis. Pullulanase specifically hydrolyzes α-1,6 linkages^37^, while neopullulanase works at α-1,4 linkages^38^ (Fig. 2a). However, these two genes were found to be inactive in pure culture and co-culture, as verified by reverse transcription–polymerase chain reaction (PCR) (Supplementary Fig. 1) and real-time quantitative reverse transcription PCR (RT-qPCR; *C*_t_ value > 40). Strain A1 genome also harbors an α-amylase (DA68_13065, encoded by *amyA*) (Fig. 2a), which hydrolyses α-1,4 linkages and breaks large, insoluble starch to form soluble starch to subsequently produce smaller α-1,4 or α-1,6 oligosaccharides. However, only α-1,4 oligosaccharides can be further hydrolyzed by α-amylase to produce maltose and ultimately glucose^39^ (Fig. 2c). Comparatively, strain B1 only possesses a glucoamylase (DA69_13810) that cleaves glucose units from the non-reducing end of amylase and amylopectin by hydrolyzing α-1,4 and α-1,6 linkages at a low rate^10,40^ (Fig. 2b, c). Thus the complementary functions of the two enzymes might explain why the co-culture showed higher starch utilization rate (Fig. 1b) and amylolytic enzyme activity (Fig. 1e).

For hydrogen production, the genome of strain A1 has one cassette encoding pyruvate formate-lyase (DA68_25815–25820) and two cassettes encoding formate dehydrogenase (DA68_13965–13990 and DA68_26295–26305) (Fig. 2a and Supplementary Table 1). The genetic information for strain A1 indicates that hydrogen is produced by formate cleavage^41^ (Fig. 2c). In comparison, strain B1 genome possesses a membrane-bound hydrogenase (DA69_04835), an NADPH-ferredoxin reductase (DA69_08100), and two NADH-ferredoxin reductases (DA69_04910 and DA69_10290) (Fig. 2b). These observations suggest that electrons are transferred to hydrogenase via reduced ferredoxin in strain B1, thus driving hydrogen production^42^ (Fig. 2c).

### Metabolite-based mutualism of the microbial consortium

**Fig. 3:**
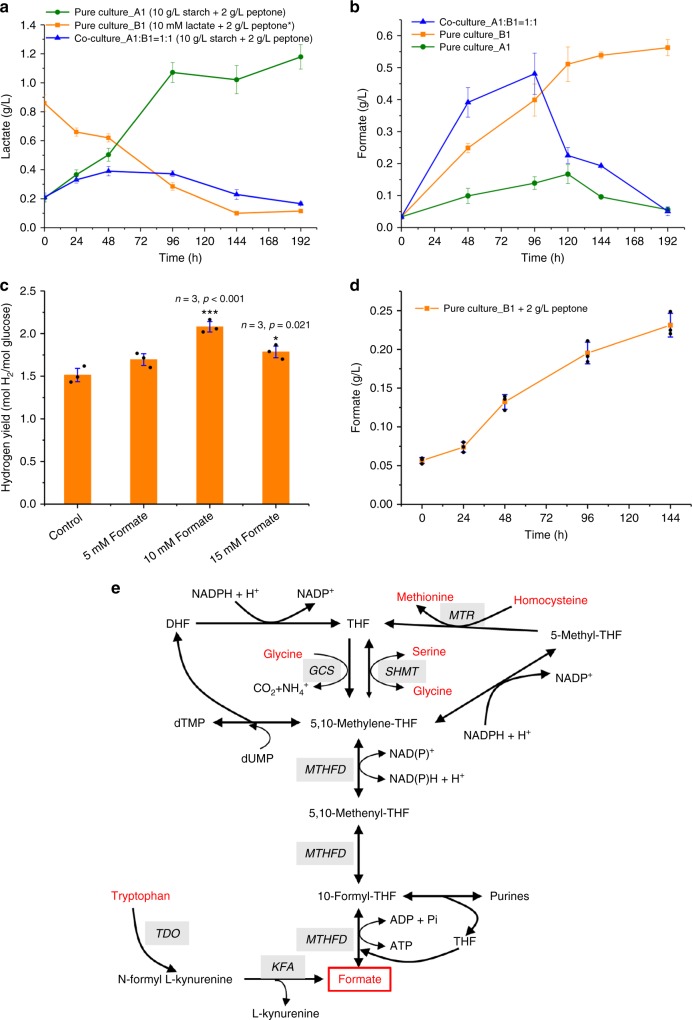
Metabolite-based mutualism in the co-culture consortium. **a** *B. cereus* A1 could convert starch to lactate, and *B. naejangsanensis* B1 could use lactate as carbon source. Asterisk (*) indicates that strains A1 and B1 cannot be cultured with inorganic nitrogen source, and peptone was added to keep the essential cell growth. **b** Formate variations in pure culture and co-culture. *B. naejangsanensis* B1 could produce formate to *B. cereus* A1 as electron shuttle for hydrogen production. **c** Addition of formate to *B. cereus* A1 enhanced the hydrogen yield. **d** *B. naejangsanensis* B1 could anaerobically produce 0.23 g L^−1^ formate using 2 g L^−1^ peptone as the only carbon and nitrogen source. **e** Formate synthesis from amino acids via folate-mediated one-carbon metabolism in *Brevundimonas naejangsanensis* B1. THF tetrahydrofolate, MTR 5-methyltetrahydrofolate-homocysteine methyltransferase, MTHFD methylenetetrahydrofolate dehydrogenase, SHMT serine hydroxymethyltransferase, GCS glycine cleavage system, TDO tryptophan 2,3-dioxygenase, KFA kynurenine formamidase. Bars indicate the average ± S.E.M of the results of three parallel replicates. Significant difference from the control group is indicated by *p < 0.05; ***p < 0.001

Metabolic interactions involving the exchange of beneficial metabolites and nutrients by bacteria are important to determine the behavior of the population in the microbial consortia^43,44^. We found that the pure culture of strain A1 could convert starch to 1.08 g L^−1^ lactate within 96 h, whereas lactate in co-culture maintained at very low level (0.17–0.39 g L^−1^, Fig. 3a), suggesting that strain B1 may utilize lactate as carbon source. To further confirm our hypothesis, we fed strain B1 with sodium lactate instead of starch. As expected, the pure culture of strain B1 could quickly consume 0.75 g L^−1^ lactate in 144 h, suggesting that strain B1 can also take lactate as carbon source (Fig. 3a).

In addition to lactate, we found that the formate concentration in the pure culture of strain A1 was low (<3.6 mM) for the entire duration of fermentation (Fig. 3b) because strain A1 can convert formate to hydrogen via formate dehydrogenase (Fig. 2a). Interestingly, pure culture of strain B1 could accumulate 0.56 g L^−1^ (12.2 mM) formate, which is likely to be an electron carrier to strain A1 for hydrogen production in co-culture. As shown in Fig. 3b, formate was accumulated up to 0.48 g L^−1^ within 96 h in co-culture, but it was quickly consumed afterwards. This large decrease of formate concentration in the co-culture after 96 h also suggests that strain A1 assimilates formate for hydrogen production (Fig. 3b). Furthermore, we also tested whether hydrogen production could be enhanced with the exogenous supplement of formate in pure culture of strain A1. As shown in Fig. 3c, the hydrogen yield of strain A1 was indeed enhanced by 37.9% and 18.3% with the addition of 10 and 15 mM formate, respectively. Taken together, we suggest that strain B1 could produce formate as an electron shuttle to strain A1 and therefore increase overall hydrogen production efficiency. Further, we observed that co-culture and pure cultures showed very similar change of pH (Supplementary Fig. 2). The pH values of all three cultures dropped almost linearly in the first 24 h due to the formation of volatile fatty acids (VFAs) and then declined gradually to a final pH of 3.3–3.4. Co-culture and pure cultures produced almost same concentration of acetate (Supplementary Fig. 2), but co-culture could generate more butyrate than pure cultures without disturbing stability of pH value, probably because of the consumption of formate and lactate through the aforesaid mutualistic interactions.

It should be noticed that strain B1 does not carry any pyruvate formate-lyase genes, implying that formate may not be generated from pyruvate. Further experiments found that, without starch supply, the pure culture of strain B1 was able to produce 0.23 g L^−1^ formate using 2 g L^−1^ peptone as the only source of carbon and nitrogen (Fig. 3d), indicating that the formate may be produced by the catabolism of some amino acids. Figure 3e shows the possible formate synthesis pathways from amino acids of strain B1, and the loci of related genes are indicated in Supplementary Table 2. One-carbon metabolism is a universal metabolic process involved in methylation reactions and metabolism of some amino acids^45^. Formate is one of the major metabolites produced from 10-formyl-tetrahydrofolate (10-formyl-THF) by 10-formyl-THF synthetase (DA69_06290)^46^. There are several amino acid metabolisms involved in this cycle. Serine and glycine are key sources of one-carbon groups. These two amino acids can interconvert to each other via serine hydroxymethyltransferase^47^ (DA69_03360 and DA69_05470), together with THF and 5,10-methylene-THF. Glycine can also be irreversibly cleaved into CO_2_ and NH_4_^+^ by the glycine cleavage system, coupled with the conversion from THF to 5,10-methylene-THF^48^. Furthermore, the remethylation of homocysteine to methionine is also involved in the formation of THF and one-carbon metabolism^49^. In addition to being a product of one-carbon metabolism, strain B1 could also produce formate by tryptophan catabolism^50^, where formate is removed from *N*-formylkynurenine by kynurenine formamidase (DA69_08900).

### Co-culture redirects the overall metabolic flux toward hydrogen production

**Fig. 4:**
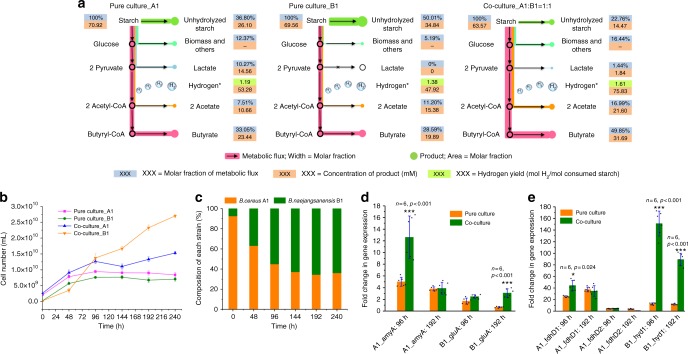
Comparison of metabolic flux, cell population, and gene transcription profiles in pure cultures and co-culture. **a** Metabolic flux distribution analysis of strains A1 and B1 in pure cultures and co-culture for hydrogen production. The fluxes of three cultures have been normalized to make total starch equal to 100%. Asterisk (*) indicates that hydrogen production does not involve in any carbon flux. **b** Cell number variations and **c** composition changes of each strain in pure cultures and co-culture by *gyrA*-based quantitative polymerase chain reaction (qPCR). The amplification efficiencies *E* for all qPCR assays were in the range of 95–97%. The regression coefficients showed a strong linear correlation (*R*^2^ > 0.991) between the *C*_t_ values and the number of cells. Transcription-level changes of genes that were involved in **d** starch utilization and **e** hydrogen production in pure cultures and co-culture at different times. *amyA* α-amylase-encoding gene (DA68_13065), *gluA* glucoamylase-encoding gene (DA69_13810), *fdhD* formate dehydrogenase-encoding gene (DA68_26295), *hyd*1 hydrogenase-encoding gene (DA69_04835). The relative expression levels were measured by reverse transcription–qPCR analysis. All data were normalized to the constant-expressed housekeeping *gyrA* gene instead of the 16S rRNA gene to eliminate the 16S rRNA similarity interferences between the two strains in co-culture samples. Significant difference between the pure culture and co-culture represent *p < 0.05; ***p < 0.001. Bars indicate the average ± S.E.M of the results of six parallel replicates

To further reveal the synergistic effects of the two strains in co-culture, the metabolic fluxes of pure cultures and co-culture were calculated based on the determination of major metabolite concentrations (Fig. 4a). In addition to hydrogen, the major metabolites of strain B1 were acetate and butyrate, while strain A1 was also capable of producing lactate. In the pure culture of A1, 36.8% of starch remained unhydrolyzed because of the limitation that α-amylase can only hydrolyze the α-1,4 linkages of starch (Fig. 2c). Meanwhile, 12.4% of starch was used for biomass accumulation and synthesis of other metabolites, and 10.3% of starch was metabolized to lactate. These pathways, however, also distributed a large proportion of carbon flux, thus reducing the hydrogen yield. The residual starch was directed into acetyl-CoA, coupled with the generation of hydrogen (1.19 mol H_2_ per mol glucose), and then acetyl-CoA was used to further produce acetate (7.5%) and butyrate (33.1%). In contrast, because glucoamylase can only hydrolyze starch into glucose units in a step-by-step manner at a low rate, 50.0% of starch was still not hydrolyzed at the end of fermentation using the pure culture of strain B1. Notably, strain B1 showed excellent hydrogen-producing properties compared to strain A1 (1.38 vs. 1.19 mol H_2_ per mol glucose), because no lactate was produced and only 5.2% starch was used for biomass synthesis. Therefore, more carbon flux was channeled to acetyl-CoA, resulting in a higher yield of hydrogen. In the co-culture, starch utilization was further enhanced to 77.2% because of the synergistic effects of the two strains. Only 1.5% of the flux was directed to lactate in the co-culture despite the fact that more starch was hydrolyzed and used, which further proved that strain B1 could use lactate as a carbon source and an electron donor, thereby redirecting the flux to hydrogen production (1.61 mol H_2_ per mol glucose).

### Co-culture enhances cell growth of both strains

Bacterial composition plays important roles in consortium functions^44,51^. In this study, a highly species-specific, DNA gyrase subunit A gene (*gyrA*)-based, quantitative PCR (qPCR) method was used in this study to quantify the changes to the bacterial composition in the pure and co-culture systems (Fig. 4b, c). Both strains A1 and B1 continued to grow until 96 h, reaching a maximal cell number of 9.45 × 10^9^ and 7.65 × 10^9^ mL^−1^, respectively. Subsequently, the cell numbers of strains A1 and B1 were maintained nearly constant, with only slight decrease. The number of cells of the individual species was higher in the co-culture than in the pure cultures alone (Fig. 4b). This increase in cell number can be chiefly attributed to the fact that cooperation between the two strains enhanced starch hydrolysis, thus supplying more carbon for strain growth. As a result, the final cell number of strain A1 increased by 82.2%. It was noteworthy that the growth of strain B1 was accelerated during the entire fermentation process, and the final cell number increased by 284.4%. This may be because strain A1 also produced lactate as an additional carbon source for strain B1 (Fig. 3a).

Figure 4c further shows the compositional changes of the two strains in the co-culture. Although the two strains were inoculated at a ratio of 1:1 (v/v), the actual cell numbers were quite different because of the different growth rates in the seed. Strain A1 grows much faster than strain B1; strain A1 dominated at the beginning of the fermentation, accounting for >90% of the total cell number. This high proportion of strain A1 during the early stage could quickly convert starch to lactate (Fig. 3a), thus supporting the growth of strain B1. As a result, the proportion of strain B1 quickly increased to 36.8% within 48 h, and continued to increase to 62.8%–65.4% with the increase in fermentation time. The increase in the population of strain B1, in return, could quickly digest proteins and produce formate for strain A1.

### Co-culture upregulates the key genes of starch hydrolysis and hydrogen production

Prior to the RT-qPCR experiment, a constantly expressed housekeeping gene, *gyrA*, was used to eliminate the interference caused by the sequence similarity of the 16S rRNA gene. The results showed that the *gyrA* gene was more specific than the 16S rRNA gene (Supplementary Fig. 3). RT-qPCR analysis showed that the genes related to starch hydrolysis and hydrogen production, present in strains A1 and B1, appeared to be differently upregulated in the co-culture (Fig. 4d, e). For starch utilization, the transcript level of the *amyA* gene (encoding α-amylase) was increased by 2.5-folds in the co-culture, compared to the pure culture of strain A1, before 96 h, indicating that strain A1 does play an important role in starch hydrolysis at an early stage. However, this level in the co-culture reduced to the same degree as that observed in case of the pure culture of strain A1, until formate was used up at 192 h (Fig. 4d). The transcript level of glucoamylase-encoding gene *gluA* in the co-culture showed no obvious increase during the early stages. At a later stage, however, the expression of this gene was increased by 4.7-folds in the co-culture, compared to the pure culture of strain B1.

Some bacteria are known to carry multiple formate dehydrogenase-encoding genes. For example, the *E. coli* genome encodes three formate dehydrogenases, but only the *hycE* gene (also referred as *hyd*3) is responsible for hydrogen production^41,52^. Likewise, because strain A1 harbors two formate dehydrogenase cassettes, both the *fdhD* genes (DA68_13975 and DA68_26295) were tested by RT-qPCR. It was found that the transcriptional level of *fdhD1* (DA68_13975) was low and showed no significant change in pure culture and co-culture during the whole fermentation, while *fdhD2* (DA68_26295) was highly expressed by 1.7-fold, compared to that observed for the pure culture of A1 at 96 h. However, this expression level decreased to the same degree after formate was used up at 192 h (Fig. 4e). In contrast, the transcription of *hyd1* in the co-culture was increased by 11.9- and 7.5-folds at 96 and 192 h, respectively, indicating that hydrogen production of strain B1 was enhanced in co-culture.

## Discussion

Here we undertook a major effort to illuminate the synergistic effects on hydrogen production from starch in a two-species microbial consortium. In this consortium, we found that the high fraction of strain A1 is a key factor that ensures the consortium has sufficient α-amylase to hydrolyze starch for cell growth. The initial ratio of strains A1 and B1 ranging from 3:1 to 1:1 showed a high starch utilization rate, whereas a lower fraction of strain A1 (A1:B1 = 1:2) would cause a 20.5% decrease in starch utilization rate (Fig. 1f). However, an excessively high fraction of strain A1 (A1:B1 = 3:1) may cause competition for nutrients between strains A1 and B1 and inhibit the growth of strain B1 during the whole process, which consequently decreases the hydrogen yield because strain B1 is important for starch hydrolysis and hydrogen production at later stage. Therefore, the optimal initial ratio of strains A1 and B1 should range from 2:1 to 1:1. Within this range, the consortium can spontaneously control the population dynamics to keep the consortium stable. However, an excessive high fraction of strain A1 or B1 would break the stability of the consortium.

**Fig. 5:**
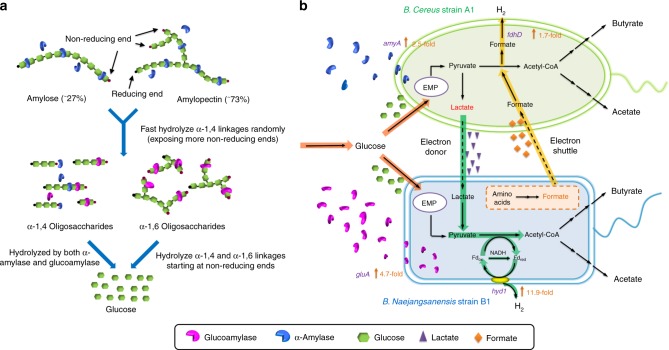
Schematic illustration of the synergistic effects between strains A1 and B1 in co-culture hydrogen production from starch. **a** Synergistic effects of α-amylase and glucoamylase on starch digestion. Strains A1 and B1 can secrete α-amylase and glucoamylase, respectively. In the co-culture, both amylose and amylopectin are rapidly hydrolyzed into low-molecular oligosaccharides, which are further hydrolyzed into glucose by the synergy of the two enzymes. **b** Metabolite-based mutualism enhances the hydrogen production. Strain A1 produces lactate for strain B1 to be used as carbon source and electron donor. Strain B1 produces formate for strain A1 to generate hydrogen. Both hydrogen production and starch hydrolysis-related genes in the two strains are upregulated accordingly. Hence, the overall hydrogen production is obviously enhanced

A microbial consortium is able to improve stability through time by controlling population dynamics of members via nutrient limitation-based effects. When nutrients become limited, a minority species can become active if it has a metabolic activity upon which survival of the entire consortium depends^53^. In our two-species consortium, this nutrient limitation-based regulation is also the key driving force for controlling population dynamics. The initial starch was determined to have 27.1% amylose and 72.9% amylopectin. Strain A1 possesses an α-amylase, which can rapidly hydrolyze both amylose and amylopectin into low-molecular-weight oligosaccharides by random cleavage of the α-1,4 glucosidic linkages (Fig. 5a), producing 63.6% α-1,4 oligosaccharides at 24 h (Supplementary Fig. 4); and then the α-1,4-linked oligosaccharides could be quickly digested into maltose and glucose. However, in the α-1,6-linked oligosaccharides, only α-1,4 linkages can be hydrolyzed, yielding only a few maltose and glucose moieties^54^. As a consequence, the percentage of α-1,4 oligosaccharides continuously decreased to 32.9% at 196 h (Supplementary Fig. 4), indicating that the starch-hydrolyzing ability of strain A1 was high at early stage but was limited when most of α-1,4 oligosaccharides were consumed. In comparison, since strain B1 only harbors a glucoamylase, glucose can be produced at a slow rate from both amylose and amylopectin by hydrolyzing the terminal or next-to-terminal α-1,4 and α-1,6 linkages, starting at the non-reducing end^10^. Importantly, hydrolyzing the α-1,6 linkages of α-1,6 oligosaccharides can also generate α-1,4 oligosaccharides, resulting in a considerable increase in the percentage of α-1,4 oligosaccharides (Supplementary Fig. 4). This makes strain B1 more important than strain A1 at later stage when most of α-1,4 oligosaccharides were hydrolyzed.

Notably, starch was converted into glucose in 20 h but was quickly utilized afterwards (Fig. 1c). During this period, the cell growth of strain A1 increased in co-culture compared with that in pure culture, while the cell growth of strain B1 was inhibited in co-culture than in pure culture (Fig. 4b), indicating that the glucose competition occurred in this stage, and strain A1 is much more competitive than strain B1. This unequal competition for glucose ensured the dominance of strain A1 to quickly digest starch at early stage. In return, strain A1 produced a large amount of lactate (Fig. 3b), which can be used by strain B1 as carbon source to support their growth and hydrogen production. As a result, the cell growth of strain B1 was accelerated after 48 h (Fig. 4b). Notably, the glucose concentration in co-culture remained at a very low level (13–15 mg L^−1^) after 24 h (Fig. 1c), indicating that glucose was limited. However, lactate was still accumulated until 96 h (Fig. 3d), and strain B1 became more dominant than strain A1. This nutrient limitation-based regulation led the consortium into a virtuous cycle, where strain A1 dominated at beginning so that it could take more glucose to produce more lactate and feed it back to strain B1. At later stage, strain B1 was dominant so that it could produce more glucoamylase to help strain A1 to hydrolyze more starch into glucose and to generate more lactate for strain B1. As a consequence, cell growth and hydrogen production were greatly enhanced by this cooperation.

One of the most important features of using microbial consortia is that it can perform complex tasks efficiently, which are difficult or even impossible for individual species^55,56^. Here we explored the underlying molecular mechanisms on how the two stains cooperated with each other to hydrolyze starch more efficiently than pure cultures (Fig. 5a). As we mentioned above, strain A1 dominated at early stage to secrete sufficient α-amylase that can quickly break both amylose and amylopectin into α-1,4 or α-1,6 low-molecular-weight oligosaccharides by endo-wise cleavage of α-1,4 linkages, thus exposing more non-reducing ends for glucoamylase (Figs. 2 and 5a). The presence of large number of oligosaccharides with many non-reducing ends may induce strain B1 to produce more glucoamylase, causing the upregulation of *gluA* by 4.7-folds (Fig. 4d). The α-1,4 oligosaccharides can then be quickly digested into glucose units, under the combined effect of α-amylase and glucoamylase, whereas the α-1,6 oligosaccharides can also be hydrolyzed by glucoamylase to provide additional glucose for cell growth. Furthermore, glucoamylase can peel starch molecules from the surface of starch granules by hydrolyzing the non-reducing ends, thus exposing new substrates to α-amylase^10^. Therefore, the synergistic effect of α-amylase and glucoamylase caused a considerably high level of α-1,4 oligosaccharides during the whole fermentation (Supplementary Fig. 4). In addition, α-amylase was reported to be inhibited by oligosaccharides, whereas glucoamylase can potentially reduce this inhibition by converting the oligosaccharides into non-inhibitory glucose units^10^, thus leading to a 2.5-fold increase in the expression of *amyA* in strain A1 (Fig. 4d). Consequently, co-culture of strains A1 and B1 can improve the starch utilization rate, compared to the pure cultures (Fig. 1b). Crude amylolytic enzyme activity assay showing that the co-culture allowed two times higher amylolytic activity compared to the pure cultures also provides considerable support for this hypothesis (Fig. 1e).

Members of a consortium communicate by exchanging metabolites or signals that allow them to coordinate their activities to accomplish a task more effectively^57^. In addition to the synergistic effects affecting starch hydrolysis, the two strains also benefited from each other for hydrogen production because of the metabolic interactions. As mentioned above, strain A1 could quickly convert starch into lactate, which was subsequently used by strain B1 as an additional carbon source (Fig. 3). Therefore, the cell numbers of strain B1 increased by 284.4% in the co-culture system (Fig. 4b). More importantly, provision of lactate to strain B1 enhanced hydrogen production because more fluxes and electrons were channeled to the hydrogen-producing pathway, which consequently upregulated the transcription of *hyd*1 during the fermentation process in the co-culture system (Fig. 4e). In return, strain B1 could break down peptone into some amino acids such as serine, glycine, and tryptophan and then release formate as electron carriers, whereby strain A1 could use these electrons to produce hydrogen via formate dehydrogenase (Fig. 5b). It has been reported that the high level of formate can enhance the expression of the formate dehydrogenase-encoding gene^58^. Indeed, the high concentration of formate (10.5 mM, Fig. 3b) produced by strain B1 in the co-culture caused a 1.7-fold increase in the expression of *fdhD2* of strain A1, compared to that observed for the pure culture of A1 at 96 h. However, this expression level decreased to the same degree after formate was used up at 192 h (Fig. 4e).

Because strains A1 and B1 were originally isolated from anaerobic-activated sludge in a foodwaste treatment reactor, which was highly rich in proteins, peptone had to be added to support cell survival. To eliminate the interference of peptone being as carbon source that might be responsible for the beneficial effects of co-culture, we conducted the experiments by replacing starch with glucose under same conditions. As indicated in Supplementary Fig. 5, the pure culture of strain A1 produced 1357.9 ± 32.2 mL L^−1^ hydrogen, while co-culture did not enhance hydrogen production but reduced it to 1157.9 ± 33.1 mL L^−1^. Furthermore, for most of amino acids from peptone being as carbon source, they have to be degraded into the precursors or intermediates of tricarboxylic acid (TCA) cycle (acetyl-coA, α-ketoglutarate, etc.) in order to generate ATP. However, our experiments were conducted under anaerobic conditions, where TCA cycle was almost completely inhibited. Instead of being as carbon source, amino acids are mainly used as the nitrogen source for DNA and protein synthesis, and the excessive amino acids are usually metabolized into VFAs under anaerobic conditions (such as formate, acetate, butyrate, etc.), acting as electron carriers or acceptors^59^. Thus it is reasonable to conclude that starch, not peptone, is the major factor for enhancing hydrogen production in the co-culture system.

Although our microbial consortium showed increased hydrogen production compared to pure cultures, the current hydrogen yield was still low compared to some studies. However, the mutual interactions in our microbial consortium could be further enhanced by additional optimization approaches. For example, we could get a much higher hydrogen yield and starch utilization using a cell immobilization technology^60^. More importantly, we could make optimal and rational design for a new synthetic consortium based on our understanding of molecular mechanisms underlying cell–cell interactions through system and synthetic biology approaches. Furthermore, we could even introduce new species into this consortium to accomplish more complicated tasks.

## Methods

### Strains, media, and culture conditions

*B. cereus* strain A1 and *B. naejangsanensis* strain B1 were isolated in our previous study, obtained from anaerobic-activated sludge. The seed medium contained 3 g L^−1^ beef extract, 10 g L^−1^ peptone, and 5 g L^−1^ NaCl. The fermentation medium was modified from our previously described medium^13^ and contained 10 g L^−1^ corn starch, 2 g L^−1^ peptone, 5 g L^−1^ NaCl, 1 g L^−1^ KH_2_PO_4_, and 1 g L^−1^ K_2_HPO_4_. All the reagents were purchased from Xilong Scientific Co., Ltd, Beijing, China. Each strain was first cultured in seed medium and was incubated at 37 °C for 72 h. Then the seed cultures at different mixed ratios (v/v) with total volume of 200 mL were inoculated into l.2 L reactors containing 800 mL fermentation medium. After inoculation, the reactors were flushed with argon gas 10 min (300 mL min^−1^) to provide anaerobic condition and then were placed in a thermostatic bath at mesophilic temperature (35 ± 1 °C).

### Enzyme activity assay

Total amylolytic activity was assayed by measuring the reducing sugars using the 3,5-dinitrosalicylic acid (DNS) method^61^ and was conducted at 35 °C for 30 min. The reaction mixture contained 50 mM sodium acetate buffer, pH 6, 1 mM CaCl_2_, and 0.5% (w/v) soluble starch with total reaction volume of 1.0 mL^62^. One enzyme unit (U) was defined as the amount of reducing sugars in 30 min under the specific condition above. Since the amount of proteins varied largely in the pure and co-cultures, the specific activity was defined as the enzyme unit (U) per volume of broth (mL) used in the assays to show the relative amylolytic activity.

### DNA extraction and whole-genome sequencing

Total DNAs of strains A1 and B1 were extracted with the Wizard Genomic DNA Purification Kit (Promega, Madison, WI). Whole genomes of strain A1 and B1 were sequenced by PacBio RS II sequencer (Pacific Biosciences, USA). The genome sequences of strains A1 and B1 were deposited in the Genbank with an accession numbers CP015727 and CP015614, respectively. Genome maps were constructed using GCView Server^63^.

### Flux distribution analysis

Metabolic flux distribution model involving 12 metabolites and 10 reactions (Supplementary Table 3) was developed based on previous studies^64,65^. Three key assumptions were made in calculating the metabolic fluxes: no net accumulation of intracellular intermediates, including pyruvate, reduced ferredoxin, acetyl-CoA, and butyryl-CoA; ATP supplies were sufficient by the glycolysis and biosynthesis of acetate and butyrate; and NADH remaining balanced.

The molar fraction of metabolite A was calculated as following:

1 $$F\left( {\mathrm{A}} \right) = \frac{{N_{\mathrm{S}}({\mathrm{A}})}}{{N_{\mathrm{S}}}} = \frac{{n \times N({\mathrm{A}})}}{{N_{\mathrm{S}}}},$$

where *F*(A) is the molar fraction of metabolite A, *N*_S_ is the molar concentration of total consumed glucose hydrolyzed from starch during the whole fermentation, *N*_S_(A) is the molar concentration of glucose used to produce metabolite A, *N*(A) is the molar concentration of metabolite A, and *n* is the stoichiometric coefficient determined by equation from starch to metabolite A according to Supplementary Table 3.

The hydrogen yield was calculated as following:

2 $$Y\left( {{\mathrm{H}}_2} \right) = \frac{{N({\mathrm{H}}_2)}}{{N_{\mathrm{S}}}},$$

where *Y*(H_2_) is the hydrogen yield, *N*_S_ is the molar concentration of total consumed glucose hydrolyzed from starch during the whole fermentation, and *N*(H_2_) is the molar concentration of hydrogen.

### Determination of relative transcriptional levels

Total RNAs of different samples were extracted with an RNAprep pure Cell/Bacteria Kit (Tiangen, Beijing, China). The RNAs was treated with DNase I (Invitrogen, CA, USA) and then was reverse transcribed to cDNA using random hexamer primers and SuperScript III reverse transcriptase (Invitrogen, CA, USA). The cDNA was used as template for qPCR analysis using the CFX96 Real-Time PCR Detection system (Bio-Rad, CA, USA) with SYBR Green RealMasterMix (Tiangen, Beijing, China). Specific primers were designed with the Beacon Designer software and are listed in Supplementary Table 4. The threshold cycle (*C*_t_) values for each gene were normalized to the reference gene *gyrA*. Amplification efficiency (*E*) of all genes are shown in Supplementary Table 5 and used to verify the specificity of the PCR products.

### Determination of cell numbers by qPCR

Cell number of each strain was determined according to the method described previously^66^. Briefly, for each strain, different ten-fold dilutions of the genomic DNA were used as template for qPCR with the primers of *gyrA* gene. Then a standard curve between the concentration of the diluted DNA and the *C*_t_ values was constructed. Since each genome has only one copy of *gyrA* gene, the copy number of *gyrA* gene is equal to the cell number. The *gyrA* gene copy numbers of the DNAs was calculated according to the equation. Number of copies per microliter = DNA concentration (μg μL^−1^) × 10^6^ (pg μg^−1^) × (1 pmol/660 pg × genome size. (bp)) × 6.022 × 10^23^ (copies mol^−1^) × 10^–12^ (mol pmol^−1^), where the genome sizes of strains A1 and B1 were determined to be 5.55 and 2.83 Mb, respectively.

### Analytical methods

Hydrogen and organic acids were determined by gas chromatography (GC-2014C, Shimadzu, Kyoto, Japan), equipped with a thermal conductivity detector, as detailed in a previous study^67^. The ratio of α-1,4/1,6 oligosaccharides was determined with the Amylose/Amylopectin Assay Kit (Megazyme, Wicklow, Ireland). Organic acids and glucose were analyzed by high-performance liquid chromatography with refractive index and ultraviolet detectors and a Bio-Rad Aminex HPX-87H column. The analysis was performed using 5 mM sulfuric acid as the mobile phase at 55 °C with a flow rate of 0.5 mL min^−1^. The total starch concentration was determined by the DNS method.

### Statistical analysis

Data analysis was performed with SigmaStat 3.5 and Excel. One-way analyses of variance were used to determine the significance of differences between groups, and *p* < 0.05 was considered as significant.

### Reporting summary

Further information on experimental design is available in the Nature Research Reporting Summary linked to this article.

## Supplementary information

Supplementary Information

Reporting Summary

## Data Availability

The genome sequences of strains A1 and B1 were deposited in the Genbank with accession numbers of CP015727 and CP015614, respectively. The authors declare that all the other data supporting the findings of this study are available within the article and its supplementary information files and from the corresponding author upon request.
