# Supplementary material for: Metabolite-based mutualism enhances hydrogen production in a two-species microbial consortium
Source: Commun Biol. 2019 Feb 28;2:82. doi: 10.1038/s42003-019-0331-8 (PMC6395672; doi:10.1038/s42003-019-0331-8)
Supplement: Supplementary file 1 — Supplementary Information [file 42003_2019_331_MOESM1_ESM.pdf]

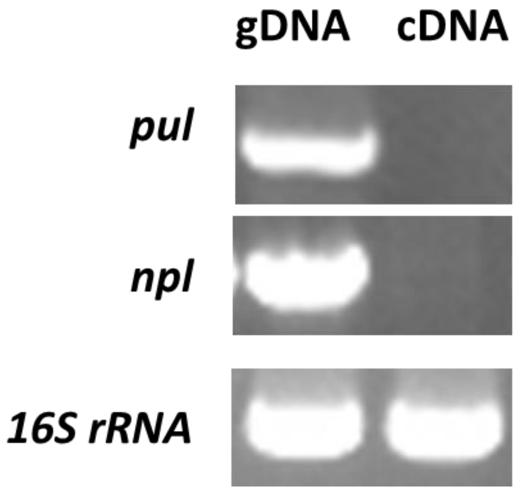

Supplementary Fig. 1. Semi-quantitative RT-PCR analysis of target gene transcripts produced in *B.cereus* A1 in mixed culture fermentation. The expression of 16S rRNA gene was used as an internal control. *pul*, pullulanase-encoding gene; *npl*, neopullulanase-encoding gene. gDNA: genomic DNA of strain A1 as a positive control; cDNA, DNA production from mRNAs of strain A1 by reverse transcription.

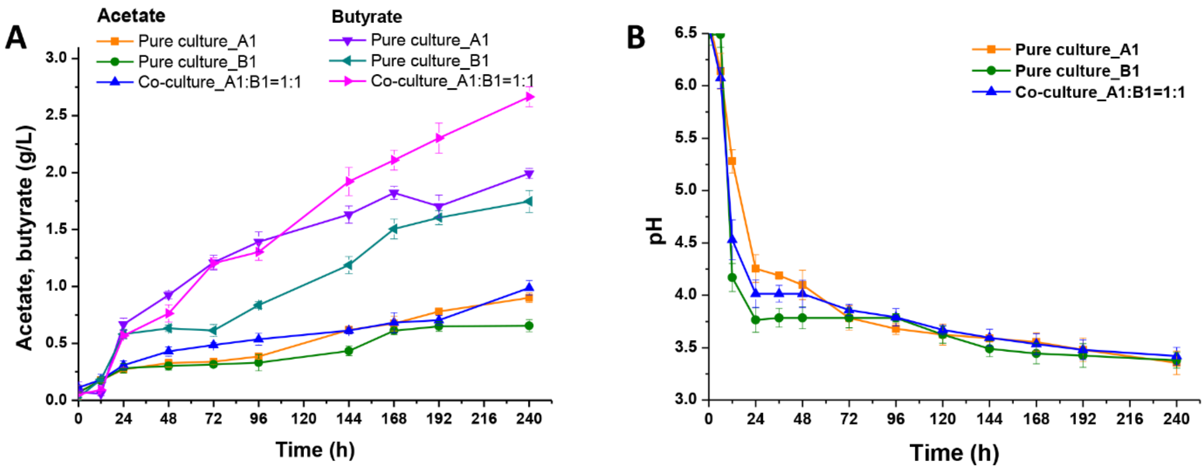

Supplementary Fig. 2. Comparison of (A) acetate and butyrate concentration and (B) pH variation between pure cultures and co-culture at a mixed ratio of A1:B1 = 1:1. Error bars indicate standard deviations from three repeat experiments.

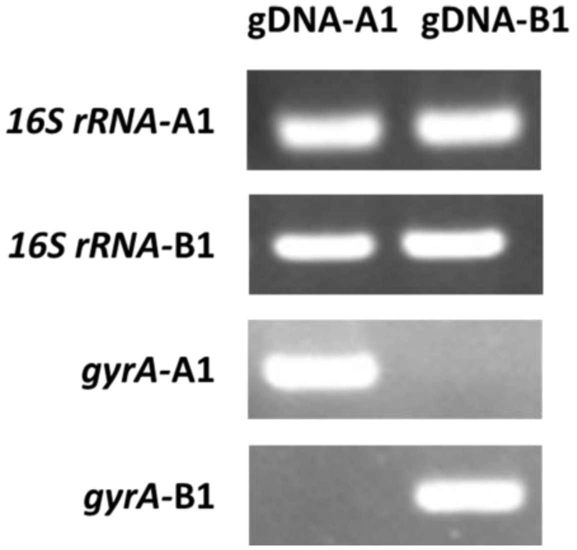

Supplementary Fig. 3. Specificity analysis of the different qPCR primers designed in this study. gDNA-A1 (B1), the genomic DNA of strain A1 (B1) as the template for PCR amplification. *gyrA*, primers designed for the amplification of gyrase subunit A gene. The result indicates *gyrA* gene shows a better specificity than *16S rRNA* gene.

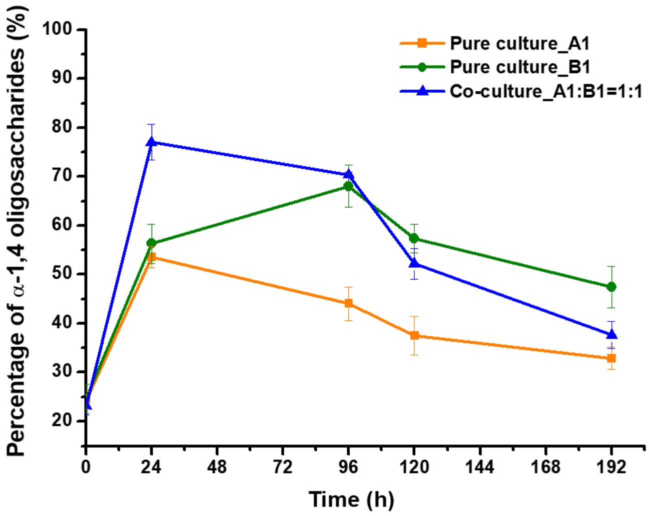

Supplementary Fig. 4. Percentage of α-1,4 oligosaccharides variation between pure cultures and co-culture at a mixed ratio of A1:B1 = 1:1. Error bars indicate standard deviations from three repeat experiments.

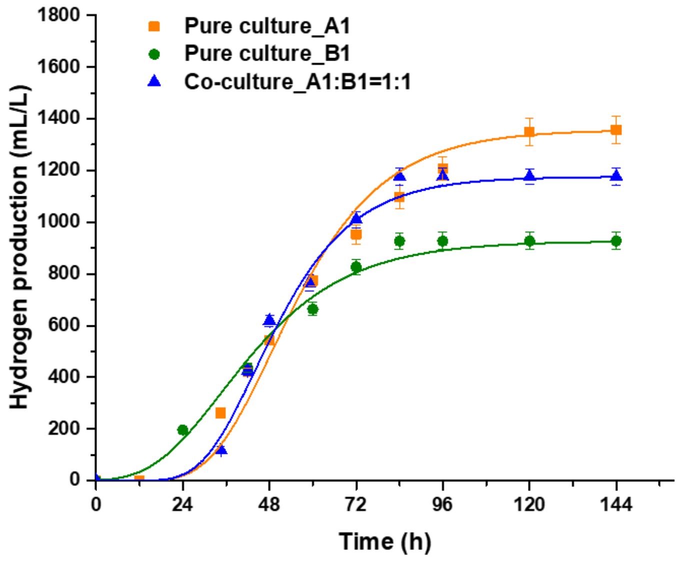

Supplementary Fig. 5. Hydrogen production from glucose between pure cultures and co-culture at a mixed ratio of A1:B1 = 1:1. The experiments were conducted under the same conditions of starch-based hydrogen production. Error bars indicate standard deviations from three repeat experiments.

Supplementary Table 1 Starch hydrolysis and hydrogen production related genes in strains A1 and B1.

Strain	Description	Locus	Gene product	Abbreviation
B.cereus A1	Starch utilization	DA68_09615	Pullulanase	*pul*
		DA68_13065	α-Amylase	*amyA*
		DA68_16145	Neopullulanase	*npl*
	Hydrogen production	DA68_13965	Formate efflux transporter	-
		DA68_13970	Molybdenum cofactor biosynthesis protein MoaA	-
		DA68_13975	Formate dehydrogenase	*fdhD1*
		DA68_13980	Hypothetical protein	-
		DA68_13985	Hypothetical protein YrhD	-
		DA68_13990	Formate dehydrogenase subunit alpha	-
		DA68_25815	Pyruvate formate-lyase	*pflB*
		DA68_25820	Pyruvate formate-lyase activating enzyme	*pflA*
		DA68_26295	Formate dehydrogenase	*fdhD2*
		DA68_26300	Formate dehydrogenase subunit alpha	-
		DA68_26305	Hypothetical protein YrhD	-
B.naejangsanensis B1	Starch utilization	DA69_13810	Glucoamylase	*gluA*
	Hydrogen production	DA69_04835	Hydrogenase	*hyd1*
		DA69_04910	NADH-Ferredoxin reductase	*nfr*
		DA69_08100	NADPH-Ferredoxin reductase	*nfr*
		DA69_10290	NADH-Ferredoxin reductase	*nfr*

Supplementary Table 2 Key enzymes and gene loci related to formate synthesis from amino acids via folate-mediated one-carbon metabolism in *Brevundimonas naejangsanensis* B1.

Key enzyme*	Gene locus
MTR	DA69_03495
MTHFD	DA69_06290
SHMT	DA69_03360<br>DA69_05470
GCS	DA69_08910
	DA69_08915
	DA69_08920
	DA69_08925
TDO	DA69_10705
KFA	DA69_08900

* THF, tetrahydrofolate; MTR, 5-methyltetrahydrofolate-homocysteine methyltransferase; MTHFD, methylenetetrahydrofolate dehydrogenase; SHMT, serine hydroxymethyltransferase; GCS, glycine cleavage system; TDO, tryptophan 2,3-dioxygenase; KFA, kynurenine formamidase.

Supplementary Table 3 Metabolic stoichiometric equations used in metabolic flux analysis for hydrogen production by strains A1 and B1.

#	Species	Equation	Strain
1	Glucose	Starch + n H_2O → n Glucose	A1 and B1
2	Pyruvate	Glucose + 2 ADP + 2 NAD^+ → 2 Pyruvate+2 NADH + 2 ATP	A1 and B1
3	Lactate	Pyruvate + NADH ↔ Lactate + NAD^+	Strain A1
4a	Acetyl-CoA	Pyruvate + CoA → Acetyl-CoA + Formate	Strain A1
4b		Pyruvate + CoA + Fd_{ox} → Acetyl-CoA + CO_2 + Fd_{red}	Strain B1
5a	Hydrogen	Formate → CO_2 + H_2	Strain A1
5b		Fd_{red} + 2 H^+ → Fd_{ox} + H_2	Strain B1
6	NADH	Fd_{red} + NAD^+ ↔ Fd_{ox} + NADH + H^+	Strain B1
7	Acetyl-CoA	Pyruvate + CoA → Acetyl-CoA + Formate	Strain A1
8	Acetate	Acetyl-CoA + ADP + Pi → Acetate + ATP + CoA	A1 and B1
9	Butyryl-CoA	2 Acetyl-CoA + 2 NADH → Butyryl-CoA + 2 NAD^+	A1 and B1
10	Butyrate	Butyryl-CoA + ADP + Pi → Butyrate + ATP + CoA	A1 and B1

Reversible reactions are indicated with '↔', whereas irreversible reactions are indicated with '→'. Fd_{ox}, oxidized ferredoxin; Fd_{red}, reduced ferredoxin.

Supplementary Table 4 Sequences of primers used in this study.

Primer	Sequence (5’-3’)
A1-*plu*-F	TGAACCGCAATATAACTG
A1-*plu*-R	ATACTACATCCATCACTACT
A1-*amyA*-F	ATCCTACACTTGCGGTTA
A1- *amyA* -R	GGCTCACTACAGATTCCA
A1-*fdhD*1-F	AGAGAATGGTGAGAAGTC
A1- *fdhD*1-R	TTAGGCATATACGGTGTAA
A1-*npl*-F	GCATATAGAGGACAGCAT
A1-*npl*-R	CCATACACTATCAATATCTTCAT
A1-*fdhD*2-F	GCAACAATCTTCTACTACAT
A1-*fdhD*2-R	GTCTTCCAATATCCATTCTT
A1-*gyrA*-F	GGCGTTATTATTCGTATGC
A1-*gyrA*-R	CCTCTTGGTCTTCCTCTA
A1-16S-F	GTTTACGGCGTGGACTACCA
A1-16S-R	CGAAGGCGACTTTCTGGTCT
B1-*hyd1*-F	ATCATCCTAATCGTTCTTCAC
B1-*hyd1*-R	GATCATGTGCGAGAGGAC
B1-*gluA*-F	CAATCACATCCGCTATCT
B1-*gluA*-R	CATCTCTTCTTCCAGACG
B1-*gyrA*-F	ATCGAAGAGATCCGCAAG
B1-*gyrA*-R	ATCAGGGTCTGCTTGTTC
B1-16S-F	CCGAAGGGCACATTCGGTAT
B1-16S-R	CTCTTCGGAGTTAGTGGCGG

Supplementary Table 5 Amplification efficiencies of key genes in strains A1 and B1 for RT-qPCR.

Strain	Gene	Slope*	R^2	E**
Bacillus cereus A1	*gyrA*	-3.446	0.992	95.3%
	amyA	-3.414	0.995	96.3%
	fdhD1	-3.396	0.997	97.0%
	fdhD2	-3.429	0.992	95.7%
Brevundimonas naejangsanensis B1	*gyrA*	-3.438	0.991	95.4%
	gluA	-3.348	0.996	98.9%
	hyd1	-3.376	0.998	97.8%

*Slope is generated from the standard curve of C_t *vs.* log DNA dilution;

**Amplification efficiency E (%) = -1+10^(-1/slope) × 100%. E value ranging from 90%-105% is considered as high amplification efficiency for qPCR.
