# Supplementary material for: Metabolite-based mutualism enhances hydrogen production in a two-species microbial consortium
Source: Commun Biol. 2019 Feb 28;2:82. doi: 10.1038/s42003-019-0331-8 (PMC6395672; doi:10.1038/s42003-019-0331-8)
Supplement: Supplementary file 2 — Reporting Summary [file 42003_2019_331_MOESM2_ESM.pdf]

nature research

Corresponding author(s): Haijia Su, Ping Xu

Reporting Summary

Nature Research wishes to improve the reproducibility of the work that we publish. This form provides structure for consistency and transparency in reporting. For further information on Nature Research policies, see Authors & Referees and the Editorial Policy Checklist.

Statistical parameters

When statistical analyses are reported, confirm that the following items are present in the relevant location (e.g. figure legend, table legend, main text, or Methods section).

n/a	Confirmed	
☐	☒	The exact sample size (*n*) for each experimental group/condition, given as a discrete number and unit of measurement
☐	☒	An indication of whether measurements were taken from distinct samples or whether the same sample was measured repeatedly
☐	☒	The statistical test(s) used AND whether they are one- or two-sided<br>*Only common tests should be described solely by name; describe more complex techniques in the Methods section.*
☐	☒	A description of all covariates tested
☐	☒	A description of any assumptions or corrections, such as tests of normality and adjustment for multiple comparisons
☐	☒	A full description of the statistics including central tendency (e.g. means) or other basic estimates (e.g. regression coefficient) AND variation (e.g. standard deviation) or associated estimates of uncertainty (e.g. confidence intervals)
☐	☒	For null hypothesis testing, the test statistic (e.g. *F*, *t*, *r*) with confidence intervals, effect sizes, degrees of freedom and *P* value noted<br>*Give P values as exact values whenever suitable.*
☒	☐	For Bayesian analysis, information on the choice of priors and Markov chain Monte Carlo settings
☒	☐	For hierarchical and complex designs, identification of the appropriate level for tests and full reporting of outcomes
☒	☐	Estimates of effect sizes (e.g. Cohen's *d*, Pearson's *r*), indicating how they were calculated
☐	☒	Clearly defined error bars<br>*State explicitly what error bars represent (e.g. SD, SE, CI)*

Our web collection on statistics for biologists may be useful.

Software and code

Policy information about availability of computer code

Data collection	The genome sequences of strains A1 and B1 were subjected to automated analysis by Rapid Annotation using Subsystem Technology (RAST, http://rast.nmpdr.org/). The metabolic pathways of strains A1 and B1 were further analyzed using the Kyoto Encyclopedia of Genes and Genomes database (KEGG, http://www.genome.jp/kegg/). Genome maps were constructed using GCView Server (http://stothard.afns.ualberta.ca/cgview_server/). Quantitative PCR (qPCR) data were collected and analyzed by CFX Manager (V 2.1).
Data analysis	Data analysis was performed with SigmaStat (V 3.5) and Microsoft Excle.

For manuscripts utilizing custom algorithms or software that are central to the research but not yet described in published literature, software must be made available to editors/reviewers upon request. We strongly encourage code deposition in a community repository (e.g. GitHub). See the Nature Research guidelines for submitting code & software for further information.

April 2018

Data

Policy information about availability of data

All manuscripts must include a data availability statement. This statement should provide the following information, where applicable:

- Accession codes, unique identifiers, or web links for publicly available datasets
- A list of figures that have associated raw data
- A description of any restrictions on data availability

The genome sequences of strains A1 and B1 were deposited in the Genbank with an accession number of CP015727 and CP015614, respectively. The authors declare that all the other data supporting the findings of this study are available within the article and its supplementary information files and from the corresponding author upon request.

Field-specific reporting

Please select the best fit for your research. If you are not sure, read the appropriate sections before making your selection.

☒ Life sciences ☐ Behavioural & social sciences ☐ Ecological, evolutionary & environmental sciences

For a reference copy of the document with all sections, see nature.com/authors/policies/ReportingSummary-flat.pdf

Life sciences study design

All studies must disclose on these points even when the disclosure is negative.

Sample size	Samples were taken from at least three repeat experiments. The one-way ANOVA were used to determine the significance of differences between groups, and $p < 0.05$ was considered as significant.
Data exclusions	The authors declare that there is no data exclusions.
Replication	The authors declare that all the experimental data are repeatable.
Randomization	Bacterial colonies from transformations were selected randomly. Allocation into experimental groups is irrelevant; single colonies were propagated as bacterial cultures, which were then split and tested with various experimental conditions, where applicable.
Blinding	The data presented did not require the use of blinding.

Reporting for specific materials, systems and methods

Materials & experimental systems

n/a	Involved in the study
☒	☐ Unique biological materials
☒	☐ Antibodies
☒	☐ Eukaryotic cell lines
☒	☐ Palaeontology
☒	☐ Animals and other organisms
☒	☐ Human research participants

Methods

n/a	Involved in the study
☒	☐ ChIP-seq
☒	☐ Flow cytometry
☒	☐ MRI-based neuroimaging
